# COVID-19 and Kidney: The Importance of Follow-Up and Long-Term Screening

**DOI:** 10.3390/life13112137

**Published:** 2023-10-30

**Authors:** Vikrant Rai

**Affiliations:** Department of Translational Research, Western University of Health Sciences, Pomona, CA 91766, USA; vrai@westernu.edu; Tel.: +1-909-469-7043

**Keywords:** COVID-19, acute kidney injury, chronic kidney disease, screening, treatment

## Abstract

Renal involvement and kidney injury are common in COVID-19 patients, and the symptoms are more severe if the patient already has renal impairment. Renal involvement in COVID-19 is multifactorial, and the renal tubule is mainly affected, along with podocyte injury during SARS-CoV-2 infection. Inflammation, complement activation, hypercoagulation, and crosstalk between the kidney and lungs, brain, and heart are contributory factors. Kidney injury during the acute phase, termed acute kidney injury (AKI), may proceed to chronic kidney disease if the patient is discharged with renal impairment. Both AKI and chronic kidney disease (CKD) increase mortality in COVID-19 patients. Further, COVID-19 infection in patients suffering from CKD is more severe and increases the mortality rate. Thus, it is important to address both categories of patients, either developing AKI or CKD after COVID-19 or previously having CKD, with proper management and treatment. This review discusses the pathophysiology involved in AKI and CKD in COVID-19 infection, followed by management and treatment of AKI and CKD. This is followed by a discussion of the importance of screening and treatment of CKD patients infected with COVID-19 and future perspectives to improve treatment in such patients.

## 1. Introduction

Post-COVID-19 conditions affecting nearly 10–20% of the patients infected with COVID-19 consist of long COVID-19 syndrome, multi-organ effects of COVID-19, and side effects of treatment for COVID-19 [1]. Long COVID-19 syndrome is characterized by a constitute of symptoms, including fatigue, headache, shortness of breath, cough, palpitations, anosmia, hyposmia, cognitive dysfunction, and brain fog, to name a few [2]. The involvement of various organs, including the cardiovascular, renal, pulmonary, and nervous system, in the post-COVID-19 phase occurs in multi-organ effects and manifest as post-COVID-19 syndrome. Post-treatment effects may also affect any organ depending on the treatment given to the patient suffering from COVID-19 infection [3,4,5]. The symptoms after COVID-19 may last up to 12 months after follow-up in 80–85% of the patients. It has also been documented that the risk of developing kidney disease is high even in mild COVID-19 cases and in patients with mild to moderate symptoms who have not been admitted to the hospital [6,7]. Because of the association of COVID-19 infection with higher mortality in hospitalized patients [8], it is important to discuss the cause–effect relationship between COVID-19 and renal disease because COVID-19 patients who survive after treatment have been shown to face an increased risk of worse kidney function during post-COVID-19 infection phase leading to chronic kidney disease, apart from acute kidney injury during COVID-19 infection [1,9]. It is also important to discuss how COVID-19 contributes to acute kidney disease (AKI) or chronic kidney disease (CKD), as well as the predisposition of CKD patients to COVID-19 and its severity because COVID-19 may disproportionately affect the patients with CKD [1]. This narrative review is focused on discussing the underlying molecular aspects and pathogenesis of AKI during infection and CKD in the post-COVID period (multi-organ effects of COVID-19).

## 2. Materials and Methods

The literature search was conducted using PubMed and Google Scholar to select the original articles, randomized clinical trials, perspectives, and review articles with keywords such as COVID-19, acute kidney injury, chronic kidney disease, renal impairment, and treatment either alone or in combination. The focus was to include articles published in the last two years, but articles from 2020 to 2021 were also included if needed for reference. The articles in the English language were selected, and non-English articles were excluded during the literature search. The article selection to include in this review was based on the article title and abstract, following which the full-text articles were reviewed and included in the bibliography. In this review, our focus was to include articles reporting or reviewing human studies, while the studies in animal models were included only to support a finding or if a preclinical trial had been conducted for a drug.

## 3. Acute Kidney Injury in COVID-19

The incidence of AKI after COVID-19 infection has been reported to be 28–34% in hospitalized patients and 46–77% in patients admitted to intensive care units (ICU). Further, it has been documented that the severity of AKI was higher in ICU patients, and this incidence may vary for different stages of AKI [1]. Acute tubular injury with loss of the brush border, non-isometric vacuolar degeneration, and even frank necrosis is the most common pathology in patients with critical COVID-19. The common histological findings are of collapsing glomerulopathy, a subtype of focal segmental glomerulosclerosis characterized by global collapse of the glomerulus and hypertrophy and hyperplasia of podocytes. Collapsing glomerulopathy mainly occurs in patients with isolated AKI, those with non-severe respiratory symptoms, or those presenting with glomerular proteinuria. In distal collecting tubules and collecting ducts, an edematous expansion of the interstitial spaces occurs, while non-specific fibrosis with infiltration of lymphocytes beneath the renal capsule has also been reported [1,10,11].

**Figure 1:**
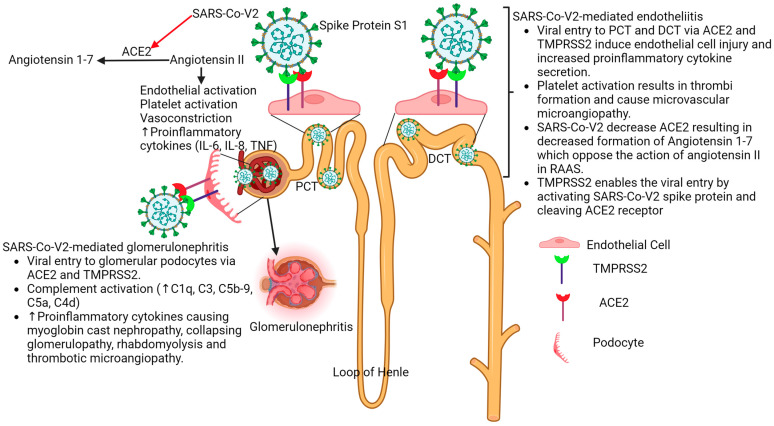
Pathophysiology of kidney injury in COVID-19 infection. Renal tubule injury with endothelial dysfunction (endotheliitis) and podocyte injury due to direct infection with SARS-CoV-2 (glomerulonephritis) occurs after viral infection. The viral entry is mediated by ACE2 and TMPRSS2 in PCT, DCT, and podocytes. This results in proteinuria, decreased urine output, and requiring KRT in severely ill patients. Angiotensin-converting enzyme 2 (ACE2), transmembrane protease, serine 2 (TMPRSS2), proximal convoluted tubule (PCT), distal convoluted tubule (DCT), complement (C), interleukin (IL), and tumor necrosis factor (TNF).

The most common pathogenesis involved in COVID-19-induced AKI is the activation of the complement cascade, acute and chronic inflammation with immune cell infiltration in the kidney, hypercoagulopathy, formation of microvascular thrombi, endothelial dysfunction (due to vascular endotheliitis), pigment nephropathy, and mitochondrial dysfunction. AKI is also caused by the direct invasion of the SARS-CoV-2 virus into podocytes and proximal convoluted tubule involving angiotensin-converting enzyme 2 (ACE2) and transmembrane protease, serine 2 (TMPRSS2) [1,11,12,13,14,15,16] (Figure 1).

**Figure 2:**
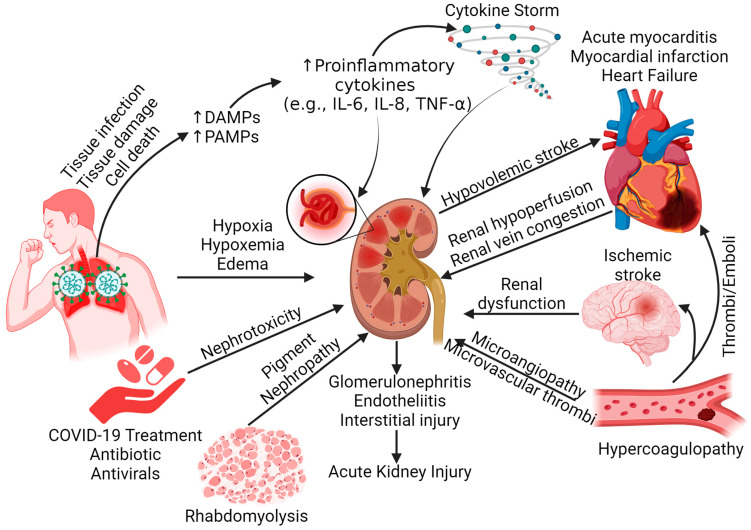
Factors contributing to the pathogenesis of kidney injury in COVID-19. Increased damage-associated molecular proteins (DAMPs) and pathogen-associated molecular proteins (PAMPs) after lung injury increase the secretion of inflammatory cytokines and contribute to kidney injury. A crosstalk between the kidney and lungs, kidney and heart, and kidney and brain also contribute to kidney injury. Hypercoagulability, pigment nephropathy, and nephrotoxic drugs contributing to kidney injury make it a multifactorial disease. Interleukin (IL) and tumor necrosis factor (TNF).

Acute respiratory distress syndrome (ARDS) may contribute to AKI through hypoxemia, increased renal vascular resistance, venous congestion, and tissue hypoxia. Tissue edema increasing renal interstitial pressure also contributes to tubular injury. The cytokines released due to circulating damage-associated molecular proteins (DAMPs) and pathogen-associated molecular proteins (PAMPs) secreted after SARS-CoV-2 infection to lung tissues and cell death contribute to local inflammation in the kidney, followed by increased recruitment of inflammatory immune cells. In addition to the crosstalk between the lung and kidney, AKI may also be mediated by a crosstalk between the cardiovascular system (renal hypoperfusion and renal vein congestion due to acute myocarditis, myocardial infarction, and heart failure) and nervous system (renal dysfunction due to ischemic stroke) (Figure 2). Additionally, direct infection of the kidney with SARS-CoV-2 induces local inflammation and damage to the kidney. Renal compartment syndrome (alteration of regional blood flow due to persistently increased pressure within a tissue), nephrotoxicity of the antibiotics (vancomycin, aminoglycosides, and colistin) and antivirals (remdesivir and ritonavir) used for treatment, and interstitial injury by infiltrating immune cells also contribute to AKI [11,12,13,14].

A study by Cantuti-Castelvetri et al. proposed that in addition to ACE2 and TMPRSS2, neuropilin1 (NRP1), a catalytic protein and a cell surface receptor, mediated SARS-CoV-2 entry and significantly potentiates SARS-CoV-2 infectivity [17]. The abundance of NPR1 in the respiratory and olfactory epithelium facilitates viral entry and increased infectivity. Through the olfactory bulb, viruses can enter the CNS, which may lead to renal dysfunction and AKI. The role of NRP1 in increasing infectivity is potentiated by the reduced SARS-CoV-2 entry and infectivity in cell culture by RNA interference or selective inhibitors [18]. Another study by Yang et al. [19] proposed kidney injury molecule-1 (KIM-1), a kidney injury biomarker and a type 1 transmembrane protein with an immunoglobulin and mucin domain, as a new host factor for SARS-CoV-2. KIM-1 binds with the SARS-CoV-2 receptor binding domain via the IgV domain and mediates viral entry. TRP channels are found in human lung tissues, and the literature suggests that TRPC6 is altered in COVID-19 infection. The alteration of TRPC6 might potentiate SARS-CoV-2-induced inflammation in patients infected with SARS-CoV-2 [20]. Further, the receptor for advanced glycation end-product (RAGE) expression is enhanced in COVID-19 and is associated with its severity and patients’ morbidity and mortality [21]. In podocytes, increased RAGE activates TRPC6 through activated Src kinases, and activated TRPC6 is related to podocyte injury [22]. Thus, TRPC6 may be a potential therapeutic target to ameliorate renal injury. This assumption was because TRPC6 inhibition with BI764198 ameliorates fibrosis and dysfunction in cardiac and renal disease [23]. However, a phase II randomized clinical trial concluded that TRPC6 inhibition with its inhibitor BI 764198 is not effective in decreasing the ARDS risk and/or severity in COVID-19 patients requiring non-invasive, supplemental oxygen support [24]. These findings may be due to a gain-of-function TRPC6 mutation leading to only a mild susceptibility to glomerular injury in mouse models [25]. CD147 may be another potential port of entry to the kidney, as CD147 is a receptor for SARS-CoV-2 [26,27].

## 4. Chronic Kidney Disease in COVID-19

AKI proceeds to CKD in the presence of chronic inflammation, cytokine storm, and the presence of factors inducing AKI. The patients developing AKI during COVID-19 infection are more likely to develop CKD compared to those who do not develop AKI (47.8% vs. 28.6%) [28]. The risk of developing CKD in COVID-19 survivors is high, and 16% of AKI may progress to CKD at 90 days; risk decreases with time with no increased risk of CKD at 12 months follow-up [12]. In addition to AKI proceeding to CKD after direct infection of SARS-CoV-2 to the kidney, CKD may also be due to delayed treatment of AKI due to the presence of a new diagnosis of cardiovascular disease or diabetes. Further, mild CKD present at the time of infection may proceed to end-stage renal disease (ESRD) needing dialysis or renal transplant. Furthermore, the presence of mild CKD predisposes a person to an increased risk of SARS-CoV-2, making a bidirectional relation between CKD and COVID-19 [29]. After COVID-19 treatment, serum creatinine levels may return to normal, but studies suggested that other molecular and cellular parameters, including the presence of inflammation, expression of various kidney genes, fibrosis, and function deficit, may persist and not return to baseline [29]. Altogether, these factors make post-COVID-19 CKD a multifactorial disease.

The substantial effect of COVID-19 on CKD is reflected by the findings of a systemic review, including 69 systematic reviews and 66 primary studies on CKD in association with COVID-19 [30]. This study reported a prevalence of 0.4 to 49% for CKD in patients with COVID-19, with a significantly increased risk of hospitalization in patients with CKD and increased mortality [30]. This study also highlighted the importance of treating CKD patients infected with SARS-CoV-2 and underscored the importance of identifying strategies to prevent SARS-CoV-2 infection in CKD patients. Regarding the prevalence of CKD in COVID-19, studies have reported a wide range depending on the geographical area of the study conducted, the stage of kidney disease, and the time of study being conducted. The prevalence of 0.84%, 2.89%, and 30% [31], from 0.5% to 30% [12], and 5.7% [32] has been reported by various studies. Another study by Bowe et al. [7] conducted included 1,726,683 patients (89,216 patients were 30-day survivors (non-hospitalized, hospitalized, and those admitted to intensive care) of COVID-19 and 1,637,467 were noninfected controls) reported a higher risk of AKI and eGFR decline in survivors of COVID-19 compared to the noninfected controls. An increased risk of kidney outcomes in the post-acute phase of COVID-19 patients suggests that such patients should be monitored for CKD. The notion that AKI proceeds to CKD is supported by the findings that patients with COVID-19 developing AKI and discharged with unresolved kidney injury are at a higher risk of mortality even after a long hospitalization [33]. Thus, patients with AKI both in the acute phase as well as after discharge should be rigorously followed up for CKD. This is important because some COVID-19 patients who are asymptomatic for renal impairment during the acute phase of infection may proceed to CKD.

## 5. Management and Treatment of AKI

As per the Kidney Disease: Improving Global Outcomes (KDIGO) guidelines, supportive care, including monitoring for renal involvement by monitoring serum creatinine and urine output and avoiding nephrotoxic drugs during SARS-CoV-2 infection in critically ill patients, may reduce the risk of developing AKI [34]. Adjustment of the fluid balance while treating the patient for hypovolemia is also important to reduce the risk of AKI by preventing fluid overload [35]. However, caution must be practiced while treating the fluid balance because hypovolemia may also lead to pulmonary embolism, mainly in COVID-19 patients with thromboembolism or with immunothrombosis. In such patients, to avoid pulmonary embolism, continuous monitoring of central venous pressure (CVP) to avoid hypotension should be practiced. [36,37,38]. Further, there is a need to investigate the mechanism of the systemic arterial pressure response, which is a cause of death by pulmonary embolism, and this is important because of the independentness of the magnitude of the pulmonary hemodynamic changes [39]. Another important aspect is the development of AKI due to mechanical ventilation and the use of diuretics in COVID-19 patients [40,41,42]. Excessive use of diuretics may cause hypovolemia, which may cause pulmonary embolism. Thus, managing hypovolemia and fluid balance is very important and should be cautiously treated. Further, using lung-protective ventilation to reduce volutrauma (lung overdistension during mechanical ventilation) and barotrauma (tissue damage due to a pressure difference during mechanical ventilation) lowers the risk of developing new AKI or worsening AKI [43]. In critically ill patients with refractory hypoxemia, early initiation of KRT prevents disease progression. Of note, in patients on KRT, along with extracorporeal membrane oxygenation (ECMO), venous access independent of the ECMO circuit should be used for KRT to decrease clot formation [44]. The detailed management plan for AKI needing kidney replacement therapy in patients with COVID-19 has been discussed by Ronco et al. [45].

The importance of aggressive and timely treatment of renal impairment in 2361 patients with AKI after COVID-19 infection (56% of the total cohort of 4221 patients) is reflected in the findings of the STOP-COVID Cohort Study [46] conducted on patients with AKI and treated with dialysis. This study reported that urine output and glomerular filtration rate at the time of initiation of kidney replacement therapy (including intermittent hemodialysis, continuous hemofiltration and hemodialysis, and peritoneal dialysis) are important determinants of recovery, and oliguria and lower baseline eGFR are strongly and independently associated with nonrecovery of kidney in seriously ill patients. These findings and the importance of timely intervention are further supported by the findings of an increased odds of rapid decline in kidney function (decreasing eGFR) in patients with COVID-19 compared to patients without COVID-19 (2.0% vs. 1.9% in a patient population of 97,203, with 9% with grade 3–4 CKD) reported by Diamantidis et al. [47].

The number of observational studies investigating renal function and following up with patients after acute COVID-19 infection is limited. A study with 12-month follow-up reported that mortality in COVID-19 patients with renal involvement is higher even with hemodialysis compared to patients with hemodialysis but without COVID-19 infection [48]. An observational study from New York reported that compared to extracorporeal dialysis, the treatment of AKI with peritoneal dialysis was not associated with worse clinical outcomes [49]. Another study reported that mortality was higher in patients with hemodialysis compared to peritoneal dialysis and renal transplant patients, while mortality was higher in peritoneal dialysis and renal transplant patients compared to the general population. This study concluded that the association of adverse outcomes in such patients with COVID-19 infection was not only due to common risk factors associated with COVID-19 but also depended on the location of the study, healthcare workers, and infected proportion in the general population [50]. However, well-controlled studies with a larger cohort of patients are needed to support these findings. Another study reported that the glomerular filtration rate significantly declines after 6 months in patients with COVID-19 and even in patients who demonstrate no AKI during acute infection [51]. This suggests that pathologies other than tubular injury may be the cause of declining renal function in the COVID-19 acute and post-acute phases. A recent study by Ramamoorthy et al. [52] reported that kidney fibrosis may be the underlying cause of renal impairment. This study examined the markers for inflammation, scarring, and damage and found increased expression of markers for scarring and inflammation after initial infection of the mice kidneys with corona-like virus (MHV-1 virus). Further, they treated the mice with a synthetic peptide (SPIKENET) and found its potential to prevent viral attachment and entry and decreased expression of markers for inflammation and scarring in both mice groups treated early and after one year. These findings suggest that kidney fibrosis begins in an early phase and may proceed to CKD if not treated, assuming there is no tubular injury after screening. Thus, all COVID-19 patients should be thoroughly screened for renal impairment and not only for higher levels of creatinine.

## 6. COVID-19 in Patients with CKD

Another important aspect to discuss is the care of patients with chronic kidney disease and those infected with SARS-CoV-2. Patients with chronic kidney conditions, including ESRD patients on KRT, have immune dysfunction and are considered immunocompromised. Such patients have an increased risk of infection, including SARS-CoV-2. A systematic review and meta-analysis by Nopsopon et al. [53] reported a higher prevalence of COVID-19 and mortality in patients with CKD and highlighted the need for a specific protocol to prevent COVID-19 infection and for the treatment to attenuate prevalence and adverse events. Another multicenter study [54] reported increased hospitalization and mortality in CKD patients suffering from COVID-19 in severe and moderate CKD, as well as in mild CKD. The same study also found that the higher risk of COVID-19 severity and mortality in patients with CKD is due to the associated comorbidities, including smoking, alcoholism, obesity, diabetes, hypertension, cerebrovascular disease, ischemic heart disease, heart failure, and chronic lung disease because the mortality risk decreases significantly after adjusting the data for these comorbidities. A persistent low-grade inflammation, an altered immune response, and increased susceptibility to direct infection to the kidney in CKD patients may be the underlying causes for increased severity and mortality.

Regarding the comorbidities, the ERA-EDTA Council and the ERACODA Working Group [55] reported that hypertension is not an independent risk factor for COVID-19, but the factors associated with the highest mortality include dialysis, organ transplantation, and CKD. Renal artery disease is a factor associated with increased mortality. Further, the risk associated with grades 4 and 5 CKD is much higher than chronic heart disease and diabetes mellitus. The urgent need for a specific protocol to treat CKD patients with COVID-19 was further highlighted [55] by Nopsopon et al. [53]. For better treatment and care of CKD patients with COVID-19, the ERA-EDTA Council and the ERACODA Working Group outlined a course of action, including mandatory screening for proteinuria/albuminuria for all COVID-19 patients, transferring all CKD patients with COVID-19 infection to higher centers for better care, investigating potential novel immune and inflammatory targets and molecular processes involved in various stages of CKD patients during COVID-19 infection, to design better triage criteria by epidemiological studies, and including such patients in vaccine and clinical trials testing drugs [55].

The notion that CKD patients requiring dialysis or not dependent on dialysis should be screened with extra care and treatment should be started with priority is further supported by the finding of the Study of Treatment and Outcomes in Critically Ill Patients With COVID-19 (STOP-COVID) [56], including 143 patients dependent on dialysis, 521 patients not dependent on dialysis, and 3600 patients without CKD. This study reported that CKD patients requiring dialysis are more likely to show altered mental status but fewer symptoms of typical COVID-19 and less likely to require mechanical ventilation. However, the mortality rate in patients with CKD dependent or not dependent on dialysis was higher than the patients without CKD, and nearly 50% of patients died within 30 days. This study emphasized that clinical trials should be conducted to include newer COVID-19 treatment strategies in patients with CKD because the higher mortality rate in CKD patients may be due to the lack of treatment with newer therapies for COVID-19. The need for more clinical trials is supported by the fact that infection with COVID-19 persists [57], though decreases due to hybrid immunity, due to infection with other predominantly circulating variants of the virus, and not by BA.2.86 (as per CDC). A randomized control trial of Ravulizumab [58], a humanized monoclonal antibody that inhibits the cleavage of C5 into C5a and C5b, in kidney patients reported improved mortality preceded by anuria in patients receiving standard care along with Ravulizumab. This study concluded the need for large-scale clinical trials, including multivariate models, to support the findings of decreased mortality. Another study reported that Remdesivir might not be nephrotoxic and that treatment of COVID-19 with Remdesivir is not associated with AKI [59]. This suggests that Remdesivir may be a potential therapeutic in CKD patients, though it warrants clinical trials.

## 7. Biomarkers of Kidney Injury in COVID-19

COVID-19 pandemic-related impact on kidney disease, both AKI and CKD, has accelerated research into the biomarkers important in diagnosing AKI early and preventatively. The pandemic has taught us that the earlier the diagnosis of AKI, the greater the probability of a better outcome for these patients because AKI and CKD are associated with higher mortality in COVID-19 patients. Table 1 summarizes the common possible biomarkers for AKI in COVID-19 patients, as reported in various studies [60,61,62,63,64,65,66]. IL-6 was found to have a moderate accuracy of prediction, while cystatin C, KIM-1, urine-Klotho, TIMP-2, and IL-6 had poor accuracy for predicting the incidence of AKI. Further, soluble programmed cell-death receptor-1 (sPDL-1), soluble intercellular adhesion molecule-1 (sICAM-1), and soluble vascular adhesion molecule-1 (sVCAM-1) were also found to be associated with severe AKI and ARF. Additionally, the levels of CCL-2, CCL-3, CCL-4, CXCL-8, CXCL-10, IFN-γ, IL-2, IL-6, TNF-α, IL-1Ra, IL-10, and VEGF were also found to be significantly higher in AKI patients [60,61,62,63,64,65,66]. The role of inflammatory cytokines as biomarkers is supported by the fact that inflammatory cytokines are increased during cytokine storm, which is associated with AKI [67]. The role of [TIMP-2]*[IGFBP7] as a biomarker is supported by the notion that after cardiac surgery, TIMP2*IGBP7 as a biomarker is used to diagnose AKI in septic patients and may be a prognostic marker for renal replacement therapy requirement [68].

## 8. Future Perspectives

Because of the involvement of the kidney in SARS-CoV-2 and its continuing infection with newer variants, including the New COVID EG.5 “Eris” Variant, it is important to monitor renal health in a person suffering from these new variants, as well as in patients who suffered from SARS-CoV-2 and other variants, whether they have developed AKI or CKD. Larger scale studies for estimating the infectivity by these variants and well-planned randomized controlled studies for better therapeutics should be conducted. Assessment of the kidney involvement may be conducted via urine analysis (proteinuria or hematuria is an early sign of kidney involvement), serum creatinine levels (increased creatinine levels), serum cystatin C levels, measuring glomerular filtration rate, and kidney biopsy while tubular injury may be assessed by urinary β2-macroglobulin levels [69].

Apart from monitoring AKI or CKD in acute and post-COVID-19 infection, it is also important to design novel therapeutics because of the involvement of multifaceted pathogenesis. A recent study by Wang et al. [70] reported that SARS-CoV-2 N protein is involved in AKI during infection via smad3-dependent G1 cell cycle arrest, and, thus, targeting Smad3 might be a novel strategy. The same group of researchers also reported that Smad3 is involved in AKI via the Smad3-Ripk3/MLKL necroptosis pathway during SARS-CoV-2 infection [71]. These findings further potentiate the notion of targeting Smad3 to treat AKI in COVID-19. As discussed above, complement activation occurs during COVID-19 infection, and complement activation is also involved in renal impairment and contributes via increased inflammation and kidney fibrosis [72]. Excessive activation of the complement 5a-signaling is associated with mitochondrial dysfunction, increased reactive oxygen species production, and inflammasome activation [73] and contributes to kidney injury. On 4 April 2023, the Food and Drug Administration (FDA) issued an Emergency Use Authorization (EUA) for Vilobelimab, an anti-C5a monoclonal antibody, for the treatment of COVID-19 (https://www.covid19treatmentguidelines.nih.gov/about-the-guidelines/whats-new/# Accessed on 23 September 2023). Since C5a contributes to kidney injury, Vilobelimab might be a potential therapeutic for CKD patients; however, its nephrotoxicity and its effects on mortality need to be investigated in CKD patients.

## 9. Conclusions

Considering the existing literature reports on the mortality in patients developing CKD after COVID-19 or patients with CKD developing SARS-CoV-2 infection, emphasis should be given to designing screening strategies so that not a single patient is neglected in their treatment. This is important because mechanical ventilation and proper treatment in mild cases in intensive care units has resulted in improved clinical outcome. Further, as discussed above, patients with kidney injury should be included in a vaccination program and newer therapies, though there is a need for future investigations with large clinical trials for the safety and efficacy of drugs. Since COVID-19 is a multifactorial disease, considering multiple targets or newer targets to decrease disease severity and kidney damage might be beneficial in improving clinical outcomes.

## Figures and Tables

**Table 1:** Emerging serum or urinary biomarkers of acute kidney injury (AKI) in COVID-19. Kidney injury molecule-1 (KIM-1), liver-type fatty acid-binding protein (L-FABP), interleukin (IL), soluble urokinase plasminogen activator receptor (suPAR) are early biomarkers in predicting AKI, following cardiac surgery and in patients in the ICU, neutrophil gelatinase-associated lipocalin (NGAL), tissue inhibitor of metalloproteinases 2 (TIMP2), insulin-like growth factor (IGF)-binding protein (IGFBP), soluble tumor necrosis factor receptor-1 (sTNFR-1), and soluble triggering receptor on myeloid cells-1 (sTREM-1).

Biomarker	Description	Why a Biomarker of AKI in COVID-19
Cystatin C	An endogenous cysteine proteinase inhibitor. Filtered through the glomerulus and then reabsorbed and catabolized in the proximal tubule completely.	Serum CysC had a high predictive value for COVID-19-related AKI. Moderately predictive of disease severity. Independently related to the risks of critical illness and mortality among COVID-19 patients.
KIM-1	A transmembrane protein with an extracellular immunoglobulin-like domain over top of a long mucin-like domain	Increased levels are associated with ischemia-, nephrotoxicants-, sepsis-, and immune-related injuries of the kidney proximal tubule. Renal KIM-1 mRNA levels increase 24-fold in patients with COVID-19 with bacterial sepsis. KIM-1 is a receptor for SARS-CoV-2 in the lung and kidney epithelium. KIM-1 may be a biomarker for early-stage AKI and predict a higher risk for clinical deterioration. Urine KIM-1/creatinine ratio is associated with COVID-19-specific death.
l-FABP	A lipid-binding protein that can be localized predominantly in the proximal tubule. A promising biomarker for kidney disorders and also attenuates renal injury.	l-FABP concentration substantially decreases in COVID-19 patients. Increasing l-FABP levels are associated with severity of disease and adverse clinical outcomes. Higher l-FABP levels are associated with death, pulmonary embolism, stroke, myocardial disease, prolonged hospitalization, and mechanical ventilation in COVID-19.
IL-18	A cytokine of IL-1 superfamily is activated by caspase-1 and subsequently secreted by renal tubular cells and macrophages.	Increased urinary level of IL-18 is an early diagnostic and prognostic marker of AKI. Serum IL-18 concentrations correlate with the severity of COVID-19.
suPAR	UPAR is a membrane-bound receptor and is cleaved in response to inflammatory stimuli.	SuPAR levels are highly increased in COVID-19 and mediate AKI. SuPAR levels are predictive of in-hospital AKI and the need for dialysis in COVID-19. UPAR is a predictor of disease progression biomarkers in COVID-19. SuPAR has prognostic utility in COVID-19 hospitalized patients to predict severe complications.
NGAL	A protein of the lipocalin family.	Urinary NGAL levels are elevated in patients developing AKI in ICU. Maximum urinary NGAL values are correlated with the length of mechanical ventilation. Can be an AKI biomarker in patients with COVID-19 and is strongly linked to AKI diagnosis and prediction of the duration of AKI and outcomes, including death, dialysis, shock, and longer hospital stay. May be the best marker to predict the probability of AKI in COVID-19 patients.
TIMP-2 and IGFBP7	TIMP2 is a protein-coding gene for protein-inhibiting metalloproteinases IGFBP7 is a protein, which regulates morphological changes in glandular cells.	Higher [TIMP-2]*[IGFBP7] levels were associated with adverse clinical outcomes, including the severity of AKI, requirement of RRT, and death. Elevated urinary [TIMP-2]*[IGFBP7] is a risk factor for AKI.
IL-6	An inflammatory cytokine	Higher levels are associated with AKI in COVID-19 patients.
Angiopoetin-1	Normally expressed by periendothelial cells and has a vascular protective effect.	Significantly higher levels of angiopoietin-1 are associated with AKI in COVID-19.
Neutrophil elastase 2	Secreted by neutrophils during inflammation and are involved in regulating chronic inflammation.	Increased neutrophil elastase 2 is associated with more severe AKI stages 2–3. Neutrophil elastase level 2 is significantly associated with markers of inflammation.
sTNFR-1	A transmembrane receptor that plays a key role in the regulation of the inflammatory pathway.	Associated with higher risk for severe AKI.
sTREM-1	A transmembrane receptor of the immunoglobulin superfamily plays a role in inflammatory response.	Associated with higher risk for severe AKI.

## Data Availability

This is a review article, and all data are included in this review article.
